# Optimization of *Rosa roxburghii* Tratt pomace fermentation process and the effects of mono- and mixed culture fermentation on its chemical composition

**DOI:** 10.3389/fnut.2024.1494678

**Published:** 2024-12-23

**Authors:** Xinxin Yi, Shuo Zhang, Duo Meng, Jian Zhang, Chencen Lai, Min Zhang, Xiaodong Sun, Haoxiang Yu, Pengjiao Wang, Xiuli Gao

**Affiliations:** ^1^State Key Laboratory of Functions and Applications of Medicinal Plants and School of Pharmaceutical Sciences, Guizhou Medical University, Guiyang, China; ^2^Center of Microbiology and Biochemical Pharmaceutical Engineering, Guizhou Medical University, Guiyang, China; ^3^Experimental Animal Center of Guizhou Medical University, Guiyang, China; ^4^Guizhou Provincial Engineering Research Center of Food Nutrition and Health, Guizhou Medical University, Guiyang, China

**Keywords:** *Rosa roxburghii* Tratt pomace, *Bacillus subtilis*, *Saccharomyces cerevisiae*, response surface methodology, chemical composition

## Abstract

**Background:**

*Rosa roxburghii* Tratt pomace (RRTP) contains valuable components like polyphenols and polysaccharides, which have high utilization value. Fermentation is an effective technique for creating beneficial nutrients that can improve the taste, appearance, and nutritional benefits of foods. Nevertheless, there is a lack of research on the alterations in chemical composition of RRTP during fermentation.

**Objective and Methods:**

This study aimed to ferment RRTP using *Bacillus subtilis* and *Saccharomyces cerevisiae* to improve its chemical composition. The optimal fermentation conditions for RRTP were determined through single-factor experiments and Box–Behnken design (BBD). Total phenols, total flavonoids, total triterpenes, ellagic acid, and vitamin C levels were higher in the fermented group with different strains and in the optimized group with mixed bacteria post-fermentation compared to the uninoculated group. Fermentation with different strains led to an increase in the ABTS radical scavenging capacity, DPPH radical scavenging capacity, and total antioxidant capacity (T-AOC) of RRTP.

**Conclusion:**

The HPLC-ESI-Q-Exactive Plus Orbitrap-MS method identified 20 compounds before fermentation and 34 compounds after optimized fermentation with mixed bacteria. The levels of polyphenols, flavonoids, and triterpenoids increased after the optimization with mixed bacteria. This research offers a potential approach to enhance the nutritional profile of RRTP and utilize it for the production of high-value food or feed materials.

## 1 Introduction

*Rosa roxburghii* Tratt (RRT) is a valuable medicinal and food resource found mainly in the southwest of China, particularly in Guizhou Province. RRT is a deciduous shrub belonging to the Rosaceae family, known for its high content of active substances such as vitamin C, polyphenols, polysaccharides, flavonoids, and organic acids. These compounds provide antioxidant, immune-regulating, anti-tumor, and blood glucose and lipid-regulating properties, making RRT highly sought after for its health benefits (1–4). As the bioactive components of RRT gain more recognition, there has been an increase in the deep processing of RRT, leading to the generation of fruit residues as by-products (5). The pomace left after juice extraction is often discarded as waste or used as fertilizer, resulting in environmental pollution and resource wastage. However, RRT pomace is rich in polyphenols and polysaccharides, with phenolics accounting for nearly 50% of the whole fruit (6, 7). These compounds have high utilization value, particularly in the fields of food and nutraceuticals due to their antioxidant and glycolipid metabolism-regulating properties (8, 9). RRTP is a highly nutritious and versatile ingredient that is ideal for creating dietary fiber. The fermented RRTP dietary fiber offers excellent physical properties such as water retention, decongestion, and oil absorption, as well as functional benefits like cholesterol, nitrite ion, and glucose adsorption (10). RRTP can be used as a functional food additive to promote intestinal health and enhancing their prebiotic effects (7). Additionally, RRTP can be utilized as a feed ingredient due to its vitamin C and flavonoid content (11), making it valuable for animal feed development research. Efforts to efficiently utilize RRTP and enhance its active ingredients could provide valuable insights for the integrated development and utilization of RRTP.

Fermentation is a powerful method for probiotics to utilize carbon sources and produce beneficial nutrients, which can enhance flavor, appearance, and nutritional properties of food products (12). *B. subtilis*, a key probiotic, is commonly used in medicine and health food industries. Fermentation with *B. subtilis* can break down proteins and enhance the nutritional value of food. Fermenting RRTP with *B. subtilis* has been shown to improve the physicochemical properties of pomace and the functional properties of dietary fiber (10). *Yeast* is another commonly used microorganism for fermenting food ingredients to enhance their nutritional content. *Yeast* fermentation not only improves the taste, aroma, and texture of foods, but also increases their nutritional value. Fermenting apple pomace with *yeast* can boost its fat, dietary fiber, and phenolic compounds content, as well as improve its overall nutritional and functional properties (13). While most RRTP fermentations are done with single strains, it has been shown that mixed fermentation, such as with blueberry pomace, can have significant health benefits by enhancing active ingredients and antioxidant activity (14). Similarly, mixing fermented red bayberry pomace can improve its beneficial components, antioxidant activity, and appearance (15). In conclusion, RRTP underwent fermentation with a combination of *B. subtilis* and *S. cerevisiae* to fully utilize the synergistic effect of mixed fermentation and maximize its nutrient content potential. Currently, there are no studies on the fermentation of RRTP using a mixture of *B. subtilis* and *S. cerevisiae*.

The fermentation process was optimized in this study using single-factor experiment and Box–BehnKen response surface test, with total phenolic content as the response variable. The goal was to examine impact of fermentation on total phenolic, total flavonoid, total triterpene, ellagic acid, vitamin C content, and antioxidant activity of the pomace. Additionally, potential active components in the pomace were identified using UHPLC-ESI-Q-Exactive Plus Orbitrap-MS. This research can serve as a guide for maximizing the utilization of *Rosa roxburghii* Tratt pomace resources and offer valuable insights for the industrial production of fermented pomace.

## 2 Materials and methods

### 2.1 *Rosa roxburghii* Tratt pomace

RRTP was provided by Guizhou Shanwangguo Health Industry Co., Ltd. (Guizhou, China) and stored at 4°C for further studies.

### 2.2 Reagents and fermentation bacteria

Rutin, gallic acid, ursolic acid, and ellagic acid were purchased from Beijing Solarbio Technology Co; Vitamin C and folin-phenol were purchased from Shanghai Yuanye Biotechnology Co; ABTS free radical scavenging ability, DPPH free radical scavenging ability, and T-AOC assay kit were purchased from Beijing Boxbio Science & Technology Co., Ltd.

High-activity dry *S. cerevisiae* was obtained from Angel *S. cerevisiae* Co., Ltd., Shanghai, China. The activation process for *S. cerevisiae* involved adding warm water and sucrose, followed by incubation in a thermostatic shaker. *B. subtilis* (GenBank ID: OR793953) was isolated from tempeh, a local specialty of Guizhou. The *B. subtilis* was inoculated in a seed medium and cultivated under specific conditions for 18 h.

### 2.3 Single-factor experiment

A single-factor experiment was conducted to study the impact of strain ratio, liquid-to-material ratio, fermentation time, fermentation temperature, and inoculum amount on the total phenol content of RRTP. The experiment used a fixed ratio of *B. subtilis* and *S. cerevisiae* (1:1), a liquid-to-material ratio of 2:1, a fermentation time of 48 h, a fermentation temperature of 31°C, and an inoculum amount of 8%. The effects of different strain ratios (*B. subtilis*:*S. cerevisiae* 1:3, 1:2, 1:1, 2:1, 3:1, 4:1), liquid-to-material ratios (1:1, 2:1, 3:1, 4:1, 5:1, 6:1), fermentation times (12, 24, 36, 48, 60, 72 h), fermentation temperatures (25, 28, 31, 34, 37, 39°C), and inoculum amounts (4, 6, 8, 10, 12, 14%) on the total phenolic content of RRTP were investigated.

### 2.4 Box–Behnken experiments

Based on the results of the single-factor experiment, a Box–Behnken experimental design was used to further explore the optimal fermentation conditions for RRTP. The effects of the four factors at three levels were measured as total phenolic content to determine the best fermentation conditions.

### 2.5 Scanning electron microscopy

Additionally, scanning electron microscopy was used to observe the microscopic morphology of RRTP before and after fermentation by *B. subtilis*, *S. cerevisiae*, and a mixed bacteria optimized group. A small amount of RRTP was evenly distributed on a sample stage coated with gold and placed under a scanning electron microscope (SU8100) for analysis.

### 2.6 Determination of chemical composition content

The fermented RRTP was dried in an oven at 50°C. Ethanol (80%, 1:10, w/v) was mixed with the fermented RRTP, and ultrasound-assisted extraction was performed three times at 400 watts (power) and 50°C for 1 h each time. The extracts were combined and recovered under reduced pressure to obtain a 0.1 g/mL solution. The concentrated extracts were stored in a refrigerator at 4°C for backup.

The total phenol, total flavonoid, and total triterpene contents were measured at 0 h, 12 h, 24 h, 36 h, and 48 h for the uninoculated, *B. subtilis*, *S. cerevisiae*, and mixed (1:1) groups, respectively. Changes in content were compared between before fermentation and the mixed bacteria optimized group. The ellagic acid and vitamin C contents of RRTP of uninoculated bacteria, *B. subtilis*, *S. cerevisiae*, mixed bacteria (1:1), and mixed bacteria optimized groups were measured.

#### 2.6.1 Measurement of total phenolic content

The Folin–Ciocalteu method (16) was used to determine the total phenolic content and modified accordingly. A UV–Vis spectrophotometer was utilized to measure absorbance at 735 nm. The TPC used gallic acid as a standard.

#### 2.6.2 Measurement of total flavonoid content

The total flavonoid content was measured using the aluminum nitrate-sodium nitrite colorimetric method as described in (17) with minor adjustments. The absorbance of the samples was measured at 510 nm using a UV–Vis spectrophotometer. The TFC used rutin as a standard.

#### 2.6.3 Measurement of total triterpene content

The total triterpene content was measured using the vanillin-ice acetic acid method (18). Absorbance measurements of the samples were conducted at 543 nm using a UV–visible spectrophotometer. The TTC used ursolic acid as a standard.

#### 2.6.4 Measurement of ellagic acid content

Ellagic acid was quantified using the Ultimate 3000 high-performance liquid chromatography system. The concentrated extract was centrifuged at 10,000 rpm for 10 min, and 2 mL of the supernatant was collected. To this, 2 mL of acidified methanol (containing 1.2 mol/L hydrochloric acid) was added, and the mass was measured. The solution was then refluxed at 85°C for 6 h, cooled to room temperature, and reweighed. The reduced weight was adjusted with methanol. The resulting solution was diluted 10 times with methanol and filtered through a 0.45 μm microporous filter membrane for ellagic acid content analysis. The chromatographic column used was PntulipsTMBP-C18 (4.6*250 mm, 5 μm), with a mobile phase of acetonitrile (A) and 0.25% formic acid in water (B). The injection volume was 10 μL, the column temperature was maintained at 35°C, and the solvent flow rate was 0.8 mL/min. The detection wavelength was set at 254 nm. The gradient elution for ellagic acid analysis was as follows: 0–2 min, 94–94% (B); 2–3 min, 94–84% (B); 3–10 min, 84–80% (B); 10–20 min, 80–94% (B).

#### 2.6.5 Measurement of vitamin C content

For the determination of vitamin C content, the concentrated extract was centrifuged at 10,000 rpm for 10 min, and the supernatant was filtered through a 0.45 μm microporous filter membrane. This filtered solution was used for the measurement of vitamin C content. Chromatographic column: PntulipsTMBP-C18 (4.6*250 mm, 5 μm), mobile phase: acetonitrile (A)-0.25% formic acid in water (B). With an injection volume of 10 μL, a column temperature of 35°C, and a solvent flow rate of 0.8 mL/min, the detection wavelength: 254 nm, the gradient elution was 0–30 min, 90–80% (B).

### 2.7 Measurement of antioxidant activity

#### 2.7.1 DPPH radical scavenging activity

The DPPH Free Radical Scavenging Ability Assay Kit was used to assess the samples’ ability to scavenge DPPH free radicals. The sample solution was diluted, mixed with DPPH working solution, and incubated in the dark for 30 min at room temperature before measuring absorbance at 515 nm. Trolox served as a positive control. DPPH radical scavenging rate (Ds%) was calculated as follows:


$$Ds\%=A_{\mathrm{blank}}-A_{\mathrm{test}}+A_{\mathrm{control}})/{A_{\mathrm{blank}}}^{*}100\%$$

#### 2.7.2 ABTS free radical scavenging activity

The ABTS assay kit was employed to determine the free radical scavenging ability of the samples. The sample solution was diluted, mixed with ABTS working solution and Reagent IV application solution, and incubated at room temperature for 6 min in the dark before absorbance measurement at 405 nm. Trolox was used as a positive control. The free radical scavenging rate (Ds%) of ABTS was calculated as follows:


$$Ds\%=A_{\mathrm{blank}}-A_{\mathrm{test}}+A_{\mathrm{control}})/{A_{\mathrm{blank}}}^{*}100\%$$

#### 2.7.3 Measurement of T-AOC

The FRAP method was utilized to evaluate the total antioxidant capacity following the T-AOC assay kit instructions. The sample solution was diluted, mixed with distilled water and FRAP working solution, incubated for 10 min, and absorbance was measured at 593 nm.

### 2.8 UHPLC-ESI-Q-Exactive Plus Orbitrap-MS analysis

The fermented RRTP extracts were analyzed using a Thermo Vanquish Horizon UHPLC system equipped with a Hypersil Gold C18 column (100 × 2.1 mm, 1.9 μm) to determine their chemical composition. The optimal gradient consisted of phase A (aqueous 0.1% formic acid solution) and phase B (formic acid acetonitrile solution, 0.1%): 0–3 min, 2–2% B; 3–23 min, 2–98% B; 23–26 min, 98–98% B; 26–26.1 min, 98–2% B; 26.1–28 min, 2–2% B. The column temperature was set at 40°C, with an injection volume of 2 μL and a flow rate of 0.3 mL/min. Mass spectrometry analysis was performed using the ESI-Q-Exactive Plus Orbitrap system in both positive and negative modes. The parameters included a vaporizer temperature of 350°C, capillary temperature of 320°C, spray voltage of 3.5/2.5 kV (+/−), Fullms-ddms2 universal method, scanning range of 100–1500 m/z, resolution of 70,000 (MS1) and 17,500 (MS/MS), and 20th, 40th, and 60th-order normalized Collision Energy (NCE). Raw data was imported into Compound Discoverer with a relative molecular mass deviation range of −5 to 5. Identification of compounds was based on accurate relative molecular mass information and fragmentation patterns obtained from Xcalibur software, as well as databases such as m/z Cloud, MoNA, m/z Vault, Masslist, and ChemSpider. An advanced identification algorithm, mzLogic, was used to enhance the confidence level of the results, along with fragmentation assisted reasoning and validation using Mass Frontier software for the identification of active ingredients in the fermented RRTP extracts.

### 2.9 Statistical analysis

Data were statistically analyzed and analyzed by analysis of variance (ANOVA) using GraphPad Prism 9.5.1 and design Expert 13.0.1 software. All experiments were performed in triplicate with values expressed as the mean ± standard deviation (SD) of the data obtained.

## 3 Results and discussion

### 3.1 Results of single-factor experiments

#### 3.1.1 Effect of strain ratio on TPC

**Figure 1:**
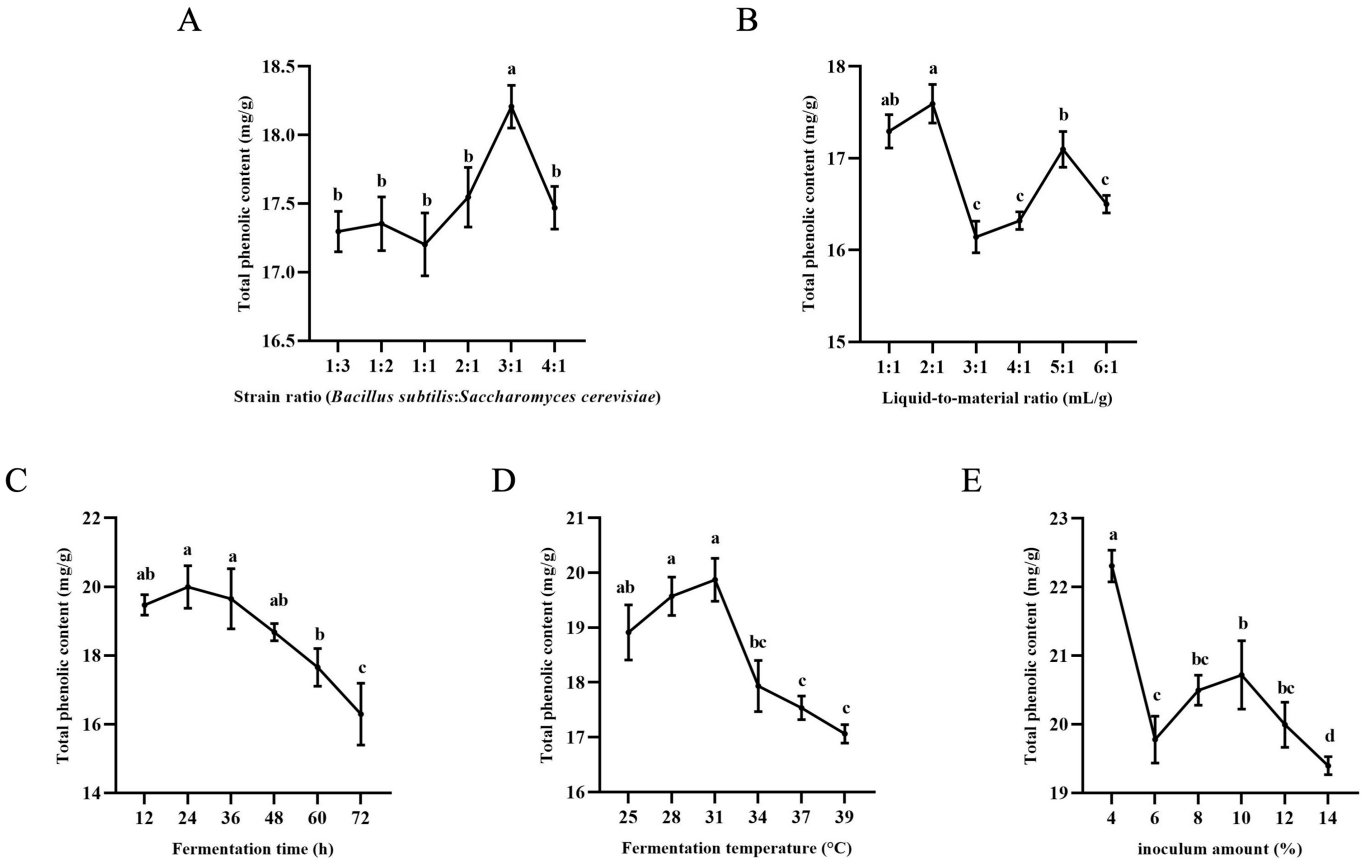
Results of single-factor experiments. **(A)** Effect of strain ratio on total phenol content. **(B)** Effect of liquid-to-material ratio on total phenol content. **(C)** Effect of fermentation time on total phenol content. **(D)** Effect of fermentation temperature on total phenol content. **(E)** Effect of inoculation amount on total phenol content. Columns marked with different lowercase letters indicate significant differences among treatments by using Duncan analysis (*p* < 0.05).

Figure 1A shows that the total phenolic content reached its peak when the ratio of *B. subtilis* to *S. cerevisiae* was 3:1. It is important to maintain proper strain ratios during fermentation to ensure optimal microbial growth and enzyme production. Lower strain ratios may lead to insufficient growth and reduced enzyme production, while higher ratios can promote rapid biomass proliferation and enzyme synthesis (19). The activity of neutral protease produced by *B. subtilis* was found to increase and then decrease with the increase in inoculum amount (20). *B. subtilis* made the fermentation process more enzyme-producing and further promoted the release of polyphenols. Therefore, the TPC was maximum when the strain ratio was 3:1. After a certain point, enzyme production may decrease due to nutrient depletion, resulting in lower polyphenol content. Therefore, the ratio of *B. subtilis* to *S. cerevisiae* was selected as 3:1 for the response surface experiment.

#### 3.1.2 Effect of liquid-to-material ratio on TPC

The total polyphenol content was found to be highest when the liquid-to-material ratio was 2:1 and lowest at 3:1, according to Figure 1B. Further increasing the liquid-to-material ratio resulted in an increase in TPC until reaching a peak at 5:1, after which TPC decreased again. This suggests that the ratio of liquid to material can impact the growth and metabolism of bacteria during fermentation. Previous studies have shown that both too low and too high moisture content can negatively affect fermentation results (21). A moisture content that is too low may not provide enough water for *B. subtilis* and *S. cerevisiae* to thrive, while a moisture content that is too high can inhibit the growth of *B. subtilis* and enzyme production, ultimately leading to a decrease in TPC (22, 23). A liquid-to-material ratio of 2:1 was selected for further optimization due to its shorter drying time and higher TPC compared to a ratio of 5:1.

#### 3.1.3 Effect of fermentation time on TPC

Figure 1C, it is observed that TPC increased from 12 to 24 h and peaked at 24 h during fermentation. However, TPC gradually decreased with prolonged fermentation time, likely due to nutrient depletion as strains require more nutrients for growth and metabolism. Phenolics may also be catabolized and metabolized as a carbon source, leading to a decrease in content. Therefore, the optimal fermentation time was determined to be 24 h.

#### 3.1.4 Effect of fermentation temperature on TPC

Temperature also plays a crucial role in enzyme activity and yield. Figure 1D demonstrates that TPC initially increases and then decreases with fermentation temperature. The highest TPC was observed at a fermentation temperature of 31°C, after which TPC decreased. This decline in TPC at higher temperatures may be attributed to the impact of elevated temperature on enzyme activity and polyphenol content. Therefore, a fermentation temperature of 31°C was chosen for subsequent experiments.

#### 3.1.5 Effect of inoculation amount on TPC

Figure 1E, it is evident that the total phenol content (TPC) was highest at a 4% inoculum level, showing an increasing trend between 6 and 10% and a decreasing trend between 10 and 14%. Excessive inoculation led to rapid metabolism in the early stages of fermentation, depleting nutrients quickly and potentially producing inhibitory metabolites that hindered the growth of strains in later stages (24). Polyphenol enrichment was found to be more effective and cost-efficient at a 4% inoculum compared to a 10% inoculum, making 4% the optimal choice.

### 3.2 Response surface method for RRTP fermentation process optimization

#### 3.2.1 Response surface test design program and results

Based on the results of the single-factor test, the strain ratio (A), liquid-to-material ratio (B), fermentation time (C), and fermentation temperature (D) were selected as the influencing factors, and total phenol content was used as the response value to conduct a Box–Behnken response surface test to optimize the fermentation process of RRTP. The Box–Behnken experimental design scheme and results are shown in Table 1.

**Table 1:** Box–Behnken design and results of tests.

Run	A: Strain ratio (*Bacillus subtilis*: *Saccharomyces cerevisiae*)	B: Liquid-to-material ratio (mL/g)	C: Fermentation time (h)	D: Fermentation temperature (°C)	Total phenolic content (mg/g)
1	3:1	1:1	12	31	25.05
2	3:1	2:1	24	31	26.71
3	4:1	2:1	24	28	23.59
4	3:1	2:1	24	31	27.79
5	3:1	1:1	24	34	26.38
6	3:1	3:1	24	34	25.24
7	2:1	2:1	24	28	22.95
8	3:1	2:1	12	34	25.47
9	3:1	1:1	24	28	24.76
10	3:1	2:1	12	28	21.90
11	3:1	2:1	24	31	28.70
12	4:1	2:1	24	34	23.69
13	2:1	2:1	24	34	24.65
14	4:1	2:1	36	31	23.60
15	3:1	2:1	36	28	24.96
16	3:1	2:1	36	34	22.24
17	3:1	3:1	36	31	24.82
18	2:1	3:1	24	31	25.72
19	2:1	1:1	24	31	25.55
20	3:1	2:1	24	31	27.05
21	4:1	2:1	12	31	24.13
22	4:1	3:1	24	31	25.49
23	3:1	1:1	36	31	25.35
24	2:1	2:1	36	31	24.67
25	4:1	1:1	24	31	25.64
26	3:1	3:1	24	28	23.00
27	3:1	2:1	24	31	29.05
28	3:1	3:1	12	31	25.80
29	2:1	2:1	12	31	23.88

#### 3.2.2 Regression modeling and analysis of variance

The results obtained in Table 1 were analyzed by regression using response surface software to obtain the multiple quadratic regression model equation for total phenol contents (Y):

Y = 27.86–0.1067A-0.2217B-0.0492C + 0.5425D-0.0800AB-0.3300AC-0.4000AD-0.3200BC + 0.1550BD-1.57CD-1.76A^2^-0.6021B^2^-1.97C^2^-2.35D^2^. According to the analysis of variance of the model (Table 2), the *p*-value of the model for total phenol content was highly significant (*p* < 0.01) and the result of the misfit term was not significant (*p* > 0.05). Therefore, the model was reliable. The R^2^ of the model was 0.9139 and adj. R^2^ was 0.8278, indicating that the model fits well and can be used to predict the results of the RRTP fermentation process. As can be seen from the *f*-values, the order of effect of the factors on the total phenol content is as follows: D (fermentation temperature) > B (liquid-to-material ratio) > A (strain ratio) > C (fermentation time).

**Table 2:** Analysis of variance.

Source	Sum of Squares	Df	Mean Square	*F*- value	*p*- value	Significance
Model	75.47	14	5.39	10.62	< 0.0001	**
A	0.1365	1	0.1365	0.2689	0.6122	
B	0.5896	1	0.5896	1.16	0.2994	
C	0.0290	1	0.0290	0.0571	0.8146	
D	3.53	1	3.53	6.96	0.0195	*
AB	0.0256	1	0.0256	0.0504	0.8256	
AC	0.4356	1	0.4356	0.8579	0.3700	
AD	0.6400	1	0.6400	1.26	0.2804	
BC	0.4096	1	0.4096	0.8067	0.3843	
BD	0.0961	1	0.0961	0.1893	0.6702	
CD	9.89	1	9.89	19.48	0.0006	**
A^2^	20.03	1	20.03	39.44	< 0.0001	**
B^2^	2.35	1	2.35	4.63	0.0493	*
C^2^	25.13	1	25.13	49.49	< 0.0001	**
D^2^	35.77	1	35.77	70.45	< 0.0001	**
Residual	7.11	14	0.5078			
Lack of fit	3.00	10	0.3003	0.2926	0.9477	
Pure error	4.11	4	1.03			
Cor total	82.57	28				

*Indicates significant differences (*p* < 0.05). **Indicates extremely significant differences (*p* < 0.01).

#### 3.2.3 Influence factor interaction analysis

**Figure 2:**
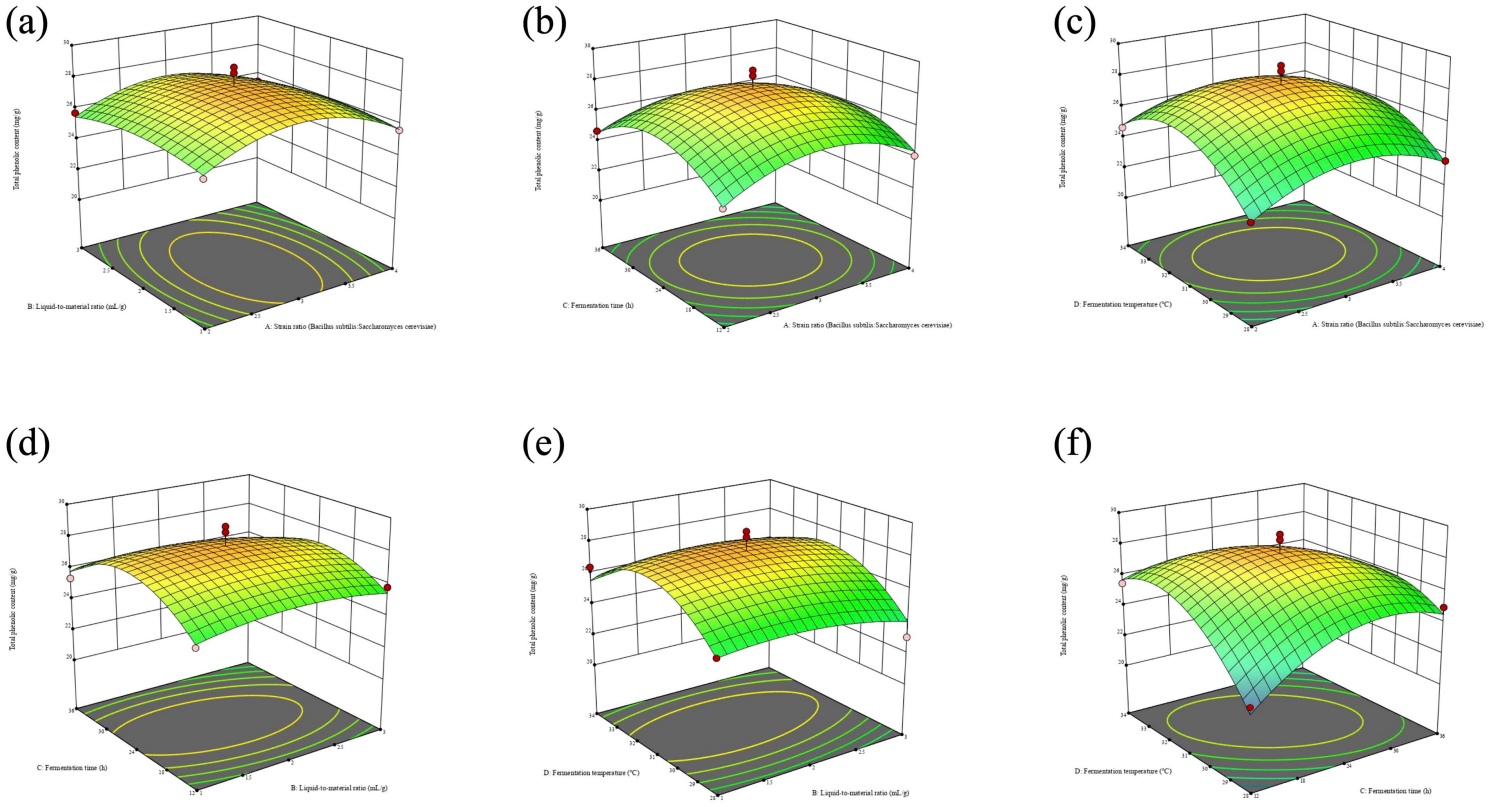
Effects of the interaction of two factors on total phenol content. **(a)** Represents the strain ratio and liquid-to-material ratio. **(b)** Represents the strain ratio and fermentation time. **(c)** Represents the strain ratio and fermentation temperature. **(d)** Represents the liquid-to-material ratio and fermentation time. **(e)** Represents the liquid-to-materia ratio and fermentation temperature. **(f)** Represents the fermentation time and fermentation temperature.

Response surface and contour plots generated from model equations visually represent the relationship between factors and response values, highlighting interaction strengths and relationships between factors (25). In contour maps, closer ellipses indicate stronger interactions, while closer circles indicate weaker interactions. The slope of the 3D response surface map indicates the magnitude of change in response values, with steeper slopes showing greater effects on response values (26). As shown in Figure 2, the steep slope of the response surface maps formed by factors AD and CD highlights the significant impact of fermentation temperature on total phenol content. The elliptical contour between Fermentation time (C) and Fermentation temperature (D) suggests an extremely significant interaction between the two factors.

#### 3.2.4 Response surface model validation tests

After conducting a fitting using Design-Expert 13.0.1 software, the optimal fermentation process for RRTP was determined. The ideal conditions included a strain ratio (*B. subtilis*: *S. cerevisiae*) of 2.962:1, liquid-to-material ratio of 1.847:1 mL/g, fermentation time of 23.413 h, and fermentation temperature of 31.390°C. This resulted in a predicted total phenol content of 27.915 mg/g. The actual fermentation process conditions were adjusted slightly for practical reasons, with a strain ratio of 3:1, liquid-to-material ratio of 2:1 mL/g, fermentation time of 24 h, and fermentation temperature of 31°C. The total phenol content measured in three parallel tests under these conditions was 27.47 ± 0.33 mg/g, which was very close to the predicted value and validated the reliability of the regression model.

### 3.3 Scanning electron microscopy analysis

**Figure 3:**
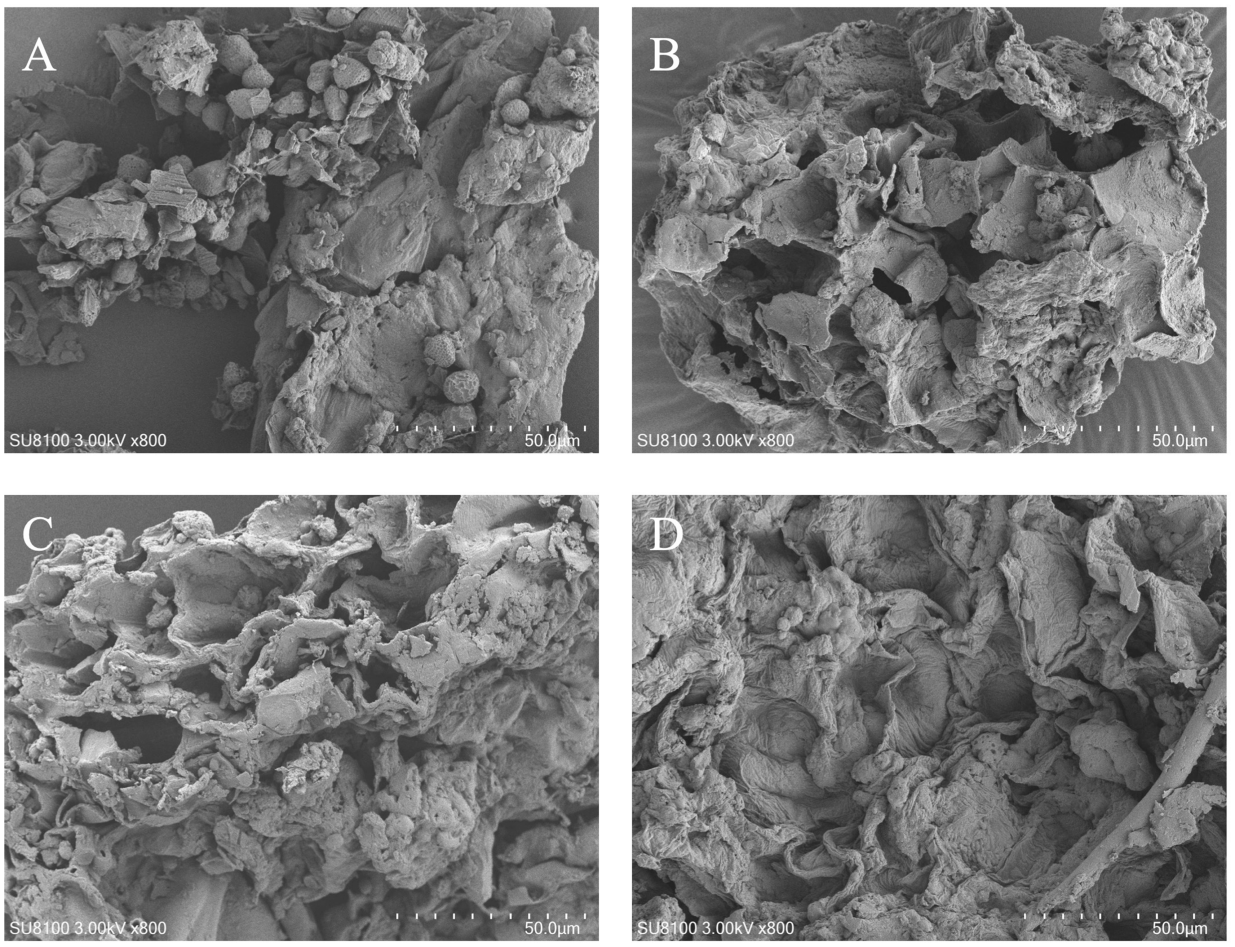
Scanning electron micrographs of RRTP before fermentation **(A)**, *B. subtilis* **(B)**, *S. cerevisiae* **(C)**, and mixed bacteria optimized for fermentation **(D)**.

Scanning electron microscopy was used to observe the microstructure of RRTP before and after fermentation with different strains (Figure 3). Prior to fermentation, RRTP exhibited a dense structure with granules on the surface. After fermentation with various strains, the granules were no longer visible, and the surface of RRTP showed numerous folds with a thin and loose structure compared to its pre-fermentation state. This change in structure may be attributed to the degradation of other components during fermentation, revealing the dietary fiber structure.

### 3.4 Change in chemical composition content

#### 3.4.1 Total phenolic content

**Figure 4:**
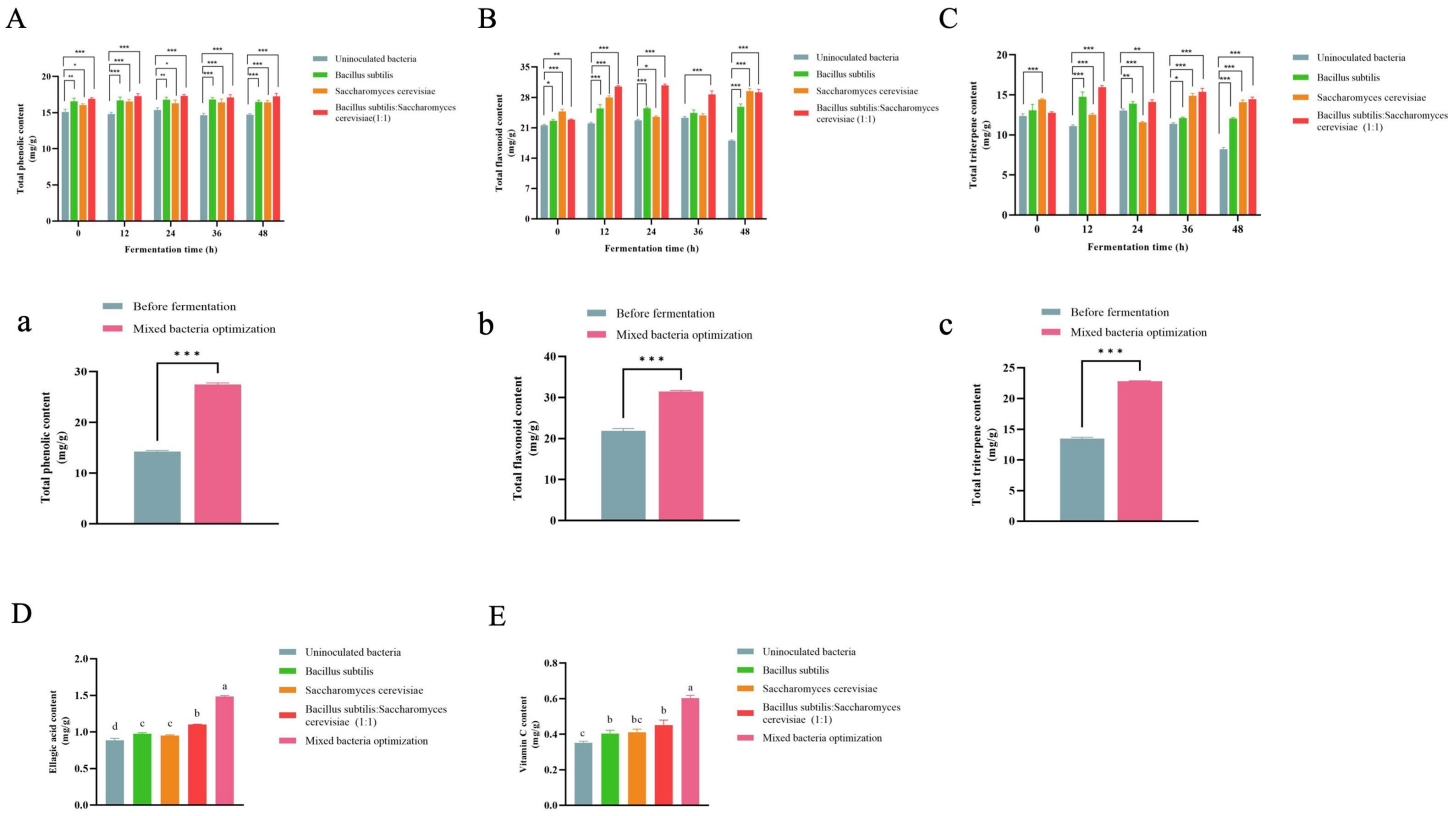
Content of different strains at different time points (*n* = 3). **(A)** Total phenol content; **(B)** Total flavonoid content; **(C)** Total triterpenoid content. Content of before fermentation and mixed bacteria optimized group. **(a)** Total phenol content; **(b)** Total flavonoid content; **(c)** Total triterpenoid content. Compared with the uninoculated bacteria group, *p < 0.05, **p < 0.01, ***p < 0.001. Content of different strain inoculation groups and mixed bacteria optimization groups (*n* = 3). **(D)** Ellagic acid content; **(E)** Vitamin C content. Columns marked with different lowercase letters indicate significant differences among treatments by using Duncan analysis (*p* < 0.05).

The fermentation process of microorganisms helps in releasing biologically active compounds from plants (27). The total phenolic content of RRTP increased significantly when fermented by different strains compared to the uninoculated group at various time points, with the highest TPC observed in *B. subtilis*: *S. cerevisiae* (1:1) among all groups (Figure 4A). After response surface optimization, the total phenol content of RRTP post-fermentation was notably higher than before fermentation (Figure 4A). It was observed that the fermentation by different strains led to an increase in TPC, with mixed strain fermentation proving to be more effective in enhancing the phenolic compounds content. This could be attributed to the hydrolysis of phenol compounds into simpler forms during fermentation, thereby exposing more phenolic hydroxyl groups (28). These findings align with those of Xi Hu et al. (29) who reported a significant increase in total phenol content in citrus pomace through probiotic fermentation.

#### 3.4.2 Total flavonoid content

The TFC of RRTP fermented by various strains was higher compared to the uninoculated group at different time points (Figure 4B). Following response surface optimization, the TFC of mixed bacteria fermentation was significantly elevated compared to pre-fermentation levels (Figure 4B), indicating an increase in TFC content due to fermentation. This increase is attributed to the release of bound flavonoids as free flavonoids (30). These results are consistent with the findings of Yan-Qiu Wang et al. (10) where the TFC of *Rosa roxburghii* Tratt pomace was found to increase post-fermentation.

#### 3.4.3 Total triterpene content

At various time points, the total triterpene content of *Rosa roxburghii* Tratt pomace (RRTP) fermented by different strains was found to be higher compared to the uninoculated group (Figure 4C). Following optimization through a response surface test, the triterpene content of mixed bacterial fermentation was significantly increased compared to before fermentation (Figure 4C), indicating that fermentation could enhance the triterpene content. These results are consistent with a study by Yanfang Yan and colleagues (18), where the triterpene content was higher after fermentation of *Rosa roxburghii* Tratt fruit by *S. cerevisiae* compared to before fermentation.

#### 3.4.4 Ellagic acid content

Ellagic acid is a potent bioactive compound that exists as a dilactone. As depicted in Figure 4D, the ellagic acid content of RRTP fermented by different strains was higher than that of the uninoculated bacterial fermentation group, and the ellagic acid content of the optimized mixed bacterial fermentation was significantly higher than the uninoculated group, indicating that fermentation could increase the ellagic acid content. Similarly, Federica Moccia et al. (31) found high levels of ellagic acid in pomegranate wastes fermented with Aspergillus niger and *S. cerevisiae*. HPLC chromatograms of ellagic acid reference substance and sample are displayed in Supplementary Figure S1.

#### 3.4.5 Vitamin C content

Figure 4E shows that the Vitamin C content after fermentation by different strains remained higher than that of the uninoculated bacterial fermentation group. Moreover, the optimization of mixed bacteria significantly increased the Vitamin C content, suggesting that fermentation can boost the Vitamin C content. This aligns with findings from Babatunde Stephen Oladeji and colleagues, who reported an increase in Vitamin C content following fermentation (32). The Vitamin C content increased post-fermentation but remained relatively low, likely due to the higher presence of Vitamin C in the fresh fruit of *Rosa roxburghii* Tratt compared to the pomace after juicing, as well as the reduction in Vitamin C content caused by drying post-fermentation. HPLC chromatograms of Vitamin C reference substance and sample are displayed in Supplementary Figure S2.

### 3.5 Antioxidant activities

**Figure 5:**
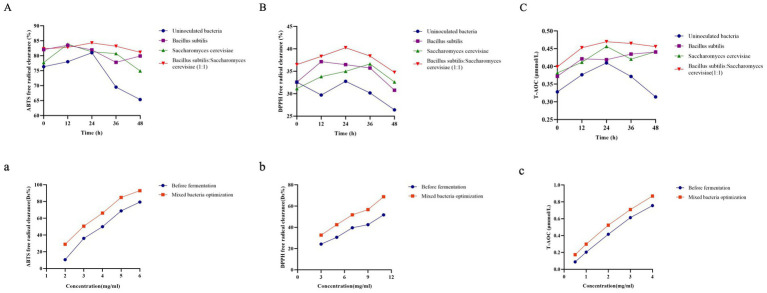
Antioxidant activity of different strains fermented at different time points (*n* = 3). **(A)** ABTS radical scavenging activity assay; **(B)** DPPH radical scavenging activity assay; **(C)** T-AOC assay. Antioxidant activity before fermentation and after optimization. **(a)** ABTS radical scavenging activity assay; **(b)** DPPH radical scavenging activity assay; **(c)** T-AOC assay.

Antioxidant activity is primarily due to the ability to scavenge free radicals (33). Figure 5 shows that RRTP fermented with inoculated bacteria exhibited higher ABTS radical scavenging capacity, DPPH radical scavenging capacity, and T-AOC compared to the uninoculated group. The antioxidant activity of RRTP was measured using three indexes before fermentation and in the mixed bacteria optimization group at different concentrations. It was observed that all the indexes of the mixed bacteria optimization group were higher than those before fermentation. The half inhibitory concentration (IC_50_ value) for scavenging DPPH radicals was 6.29 mg/mL in the mixed bacteria optimization group, while it was 11.25 mg/mL before fermentation. Additionally, the scavenging activity of ABTS radicals in the mixed bacteria optimization group (IC_50_ value of 2.9 mg/mL) was higher than before fermentation (IC_50_ value of 3.8 mg/mL). These results suggest that the antioxidant capacity improved after optimization through mixed bacteria fermentation, enhancing the antioxidant activity of RRTP. Gum et al. found that FRYP fermented with *B. subtilis* showed strong antioxidant effects and free radical scavenging ability, possibly due to its high phenolic compound content (34). Additionally, single and mixed fermentations with *S. cerevisiae* and *Lactobacillus lactis* were found to improve the antioxidant activity of fermented kiwi fruit extracts (35).

### 3.6 Preliminary identification of the chemical composition of RRTP

The initial identification of each ingredient was determined by analyzing reference substances and relevant literature. A total of 20 compounds were identified in the pre-fermentation RRTP, including 2 flavonoids, 6 terpenoids, 1 organic base, 1 sphingolipid, 1 organic acid, 3 amino acids, 2 alkaloids, and 4 other compounds. In the mixed bacteria optimized RRTP, a total of 34 compounds were identified, including 6 flavonoids, 1 phenolic acid, 2 vitamins, 8 terpenoids, 1 organic base, 1 sphingolipid, 2 organic acids, 4 amino acids, 3 alkaloids, and 6 other compounds (Table 3; Supplementary Figure S3). It was observed that flavonoids increased in the mixed bacteria optimized RRTP, possibly due to metabolism by *B. subtilis*. The addition of *B. subtilis* during fermentation has been shown to affect the release of phenolic compounds (36). The increase in terpenoids in the optimized RRTP may be attributed to the influence of *S. cerevisiae* during fermentation. Amino acids and vitamins also increased in the mixed bacteria optimized RRTP, potentially due to *S. cerevisiae*-specific metabolism. Microorganisms during fermentation not only produce organic acids to inhibit pathogenic bacteria, but also amino acids and vitamins to enhance nutritional value and functional activity (37). Compared to the pre-fermentation period, the fermentation of the optimized blend produces beneficial substances such as betaine, proanthocyanidin B1, kaempferol and ursolic acid. Betaine prevents metabolism-related fatty liver disease and its progression, as well as having neuroprotective effects, protecting myocardial function, and preventing pancreatic steatosis. Betaine also reduces oxidative stress, endoplasmic reticulum stress, inflammation and cancer development (38). Proanthocyanidin B1 is a dietary polyphenol flavan-3-ol dimer known for its antioxidant properties. Kaempferol, a common flavonoid, has various pharmacological functions including antioxidant, anti-inflammatory, and antibacterial properties (39, 40). Addition of kaempferol to broiler feed also improves ketone body characteristics by decreasing abdominal fat percentage, subcutaneous fat thickness, and muscle malondialdehyde content (41). Ursolic acid, a naturally occurring triterpenoid, also has anti-inflammatory and antioxidant effects (42). Therefore, RRTP after optimized fermentation of mixed bacteria has high medicinal and developmental value, and can be applied as a natural additive raw material in feed and other agricultural by-products.

**Table 3:** Preliminary identification of RRTP chemical constituents by UHPLC-ESI-Q-Exactive Plus Orbitrap-MS method before fermentation and after optimization of mixed bacteria.

NO.	RT (min)	Tentative identification	Molecular formular	Quasi-molecular ion	Error (ppm)	MS/MS (m/z)
Before fermentation						
1	13.717	Phytosphingosine	C_18_H_39_N_3_	318.30020[M + H]^+^	−0.85	300.29022[M + H-H_2_O]^+^, 256.26352[M + H-C_2_H_6_O_2_]^+^, 85.10129[M + H-C_12_H_27_NO_3_]^+^
2	14.096	Asiatic acid	C_30_H_48_O_5_	487.34332[M-H]^−^	0.87	470.33514[M-H-H_2_O]^−^
3	12.993	Arjungenin	C_30_H_48_O_6_	503.33835[M-H]^−^	0.91	485.32755[M - H - H_2_O]^−^
4	1.083	Choline	C_5_H_13_NO	104.10735[M + H]^+^	3.74	59.07359[M + H-N(CH_3_)_3_]^+^
5	16.504	α-Linolenic acid	C_18_H_30_O_2_	279.23169[M + H]^+^	−0.27	261.22067[M-H-H_2_O]^−^
6	11.608	Apigenin-pentoside	C_20_H_18_O_9_	401.08838[M-H]^−^	1.14	357.06213[M-H-CO_2_]^−^, 313.07205[M-H-2CO_2_]^−^
7	15.571	Glabrolide	C_30_H_44_O_4_	469.33105[M + H]^+^	0.39	451.31860[M + H-H_2_O]^+^
8	6.953	Catechin	C_15_H_14_O_6_	289.07211[M-H]^−^	0.45	179.03386[M-H-C_5_H_2_O_3_] ^−^, 137.02322[M-H-C_7_H_4_O_4_] ^−^
9	22.3	Stearamide	C_18_H_37_NO	284.29471[M + H]^+^	−0.5	130.12265[M + H-CH_2_]^+^
10	1.115	Trigonelline	C_7_H_7_NO_2_	138.05501[M + H]^+^	0.39	110.06025[M + H-CO]^+^, 94.06548[M + H-CO_2_]^+^, 79.05463[M + H-C_2_H_3_O_2_]^+^
11	3.508	L-Phenylalanine	C_9_H_11_NO_2_	166.08623[M + H]^+^	0.11	149.059888 [M + H-NH_3_]^−^, 120.080989 [M + H-COOH]^−^, 103.05454[M + H-NH_3_-COOH]^−^
12	2.104	DL-Norleucine	C_6_H_13_NO_2_	132.10197[M + H]^+^	0.53	86.09686[M + H-COOH]^+^
13	14.256	Palmitamide	C_16_H_33_NO	256.26337[M + H]^+^	−0.34	102.09174[M + H-CH_2_]^+^
14	16.649	18-β-Glycyrrhetinic acid	C_30_H_46_O_4_	469.33264[M-H]^−^	0.59	451.32245[M-H-H_2_O]^−^
15	14.609	Betulonic acid	C_30_H_46_O_3_	455.35220[M + H]^+^	0.35	439.22552[M + H-CH_3_]^+^
16	1.099	Mucic acid	C_6_H_10_O_8_	209.02992[M-H]^−^	−2.62	191.01903[M - H_2_O - H]^−^, 147.02901 [M - CO_2_ - H]^−^, 133.01302[M-H-CO_2_-CH_4_O]^−^
17	1.062	DL-Glutamine	C_5_H_10_N_2_O_3_	147.07643[M + H]^+^	0.29	131.04868[M + H-CH_2_]^+^
18	6.394	Trans-3-Indoleacrylic acid	C_11_H_9_NO_2_	188.07060[M + H]^+^	−0.09	170.05997[M + H-H_2_O]^+^, 118.06532[M + H-C_3_H_2_O_2_]^+^
19	15.939	Artemisic acid	C_15_H_22_O_2_	235.16899[M + H]^+^	−0.42	213.01106[M + H-CH_2_(OH)]^+^
20	8.493	Quercetin	C_15_H_10_O_7_	303.04971[M + H]^+^		165.01846[M + H-C_7_H_6_O_3_]^+^, 153.01831[M + H-C_8_H_6_O_3_]^+^
Mixed bacteria optimization fermentation						
1	13.685	Phytosphingosine	C_18_H_39_NO_3_	318.30093[M + H]^+^	1.21	300.29083[M + H-H_2_O]^+^, 256.26407[M + H-C_2_H_6_O_2_]^+^, 85.10191[M + H-C_12_H_27_NO_3_]^+^
2	13.935	18-β-Glycyrrhetinic acid	C_30_H_46_O_4_	471.34698[M + H]^+^	0.24	407.33151[M + H-HCOOH-H_2_O]^+^ 189.16480[M + H-C_15_H_24_O_2_-HCOOH]^+^
3	1.053	Choline	C_5_H_13_NO	104.10735[M + H]^+^	3.59	60.08139[M + H-N(CH_3_)_3_]^+^
4	12.962	Arjungenin	C_30_H_48_O_6_	503.33823[M-H]^−^	1.15	485.32809[M - H - H_2_O]^−^, 409.31705[M-H-H_2_O-COOH-CH_2_OH]^−^
5	11.579	Apigenin-pentoside	C_20_H_18_O_9_	401.08838[M-H]^−^	1.17	357.06213[M-H-CO_2_]^−^, 313.07205[M-H-2CO_2_]^−^
6	1.153	Adenine	C5H5N5	136.06177[M + H]^+^	−0.03	119.08554[M + H-NH_3_]^+^
7	1.1	Betaine	C_5_H_11_NO_2_	118.08652[M + H]^+^	2.5	59.07361[M + H-C_2_H_2_O_2_]^+^
8	12.974	Glabrolide	C_30_H_44_O_4_	469.33145[M + H]^+^	−0.13	451.32004[M + H-H_2_O]^+^
9	11.172	Corchorifatty acid F	C_18_H_32_O_5_	321.21780[M-H]-	0.32	291.19650
10	1.045	L-Glutamic acid	C_5_H_9_NO_4_	148.06049[M + H]^+^	0.04	130.05020[M + H-H_2_O]^+^, 102.05537[M + H-H_2_O-CO]^+^, 84.04488[M + H-H_2_O-CO-H_2_O]^+^, 56.05012[M + H-H_2_O-CO-H_2_O-CO]^+^
11	6.943	Catechin	C_15_H_14_O_6_	289.07220[M-H]^−^	0.51	179.03415[M-H-C_5_H_2_O_3_] ^−^, 137.02315[M-H-C_7_H_4_O_4_] ^−^
12	10.062	Kaemoferol-3-O-(6″“-o-coumaroyl)-β-D-glucoside Isomers	C_30_H_26_O_13_	593.13141[M-H]^−^	1.51	447.09549, 307.08383, 285.04019
13	22.268	Stearamide	C_18_H_37_NO	284.29471[M + H]^+^	−0.38	158.15450[M + H-C_9_H_2_0]^+^
14	20.368	Palmitamide	C_16_H_33_NO	256.26324[M + H]^+^	−0.49	102.09167[M + H-CH_2_]^+^
15	3.532	L-Phenylalanine	C_9_H_11_NO_2_	166.08624[M + H]^+^	−0.17	149.06001 [M + H-NH_3_]^−^, 120.08097 [M + H-COOH]^−^, 103.05455[M + H-NH_3_-COOH]^−^
16	1.105	Trigonelline	C_7_H_7_NO_2_	138.05505[M + H]^+^	0.72	110.06038[M + H-CO]^+^, 94.06559[M + H-CO_2_]^+^, 79.05460[M + H-C_2_H_3_O_2_]^+^
17	19.701	α-Linolenic acid	C_18_H_30_O_2_	279.23181[M + H]^+^	−0.28	261.2043[M + H-H_2_O]^+^
18	1.095	Pyrogallol	C_6_H_6_O_3_	127.03912[M + H]^+^	2.14	109.10146[M + H-H_2_O]^+^, 81.03400[M + H-H_2_O-CO]^+^, 53.03931[M + H-H_2_O-2CO]^+^
19	1.097	Mucic acid	C_6_H_10_O_8_	209.02971[M-H]^−^	−2.77	191.01897[M - H_2_O - H]^−^, 147.02882 [M - CO_2_ - H]^−^, 133.01305[M-H-CO_2_-CH_4_O]^−^
20	6.67	Procyanidin B1	C_30_H_26_O_12_	577.13635[M-H]^−^	1.18	425.08426[M-H-C_8_H_8_O_3_]^−^, 407.07761[M-H-C_8_H_8_O_3_-H_2_O]^−^, 289.07190[M-H-C_15_H_14_O_6_]^−^
21	6.377	Trans-3-Indoleacrylic acid	C_11_H_9_NO_2_	188.07057[M + H]^+^	−0.21	170.06004[M + H-H_2_O]^+^, 118.06531[M + H-C_3_H_2_O_2_]^+^
22	11.324	Betulonic acid	C_30_H_46_O_3_	455.35205[M + H]^+^	0.18	409.34763[M + H-CO-OH]^+^
23	1.523	Nicotinic acid	C_6_H_5_NO_2_	124.03949[M + H]^+^	1.32	119.95110[M-H-H_2_O-C_2_H_4_O-H_2_O]^−^
24	9.964	Abscisic acid	C_15_H_20_O_4_	263.12921[M-H]^−^	0.52	204.11488[M-H-H_2_O-C_2_HO]^−^
25	1.05	L-Homoserine	C_4_H_9_NO_3_	120.06577[M + H]^+^	2.13	102.05522[M + H-H_2_O]^+^, 84.04459[M + H-H_2_O]^+^, 74.06057[M + H-H_2_O-CO]^+^, 56.05020[M + H-H_2_O-CO-H_2_O]^+^
26	8.463	Quercetin	C_15_H_10_O_7_	303.04980[M + H]^+^	−0.41	165.01794[M + H-C_7_H_6_O_3_]^+^, 153.01823[M + H-C_8_H_6_O_3_]^+^
27	6.923	Salicylic acid	C_7_H_6_O_3_	139.03897[M + H]^+^	0.00	95.08604[M + H-CO_2_]^+^
28	15.126	Asiatic acid	C_30_H_48_O_5_	487.34348[M-H]^−^	−0.35	469.33179[M + H-H_2_O]^+^, 423.32565[M + H-H_2_O-HCOOH]^+^
29	1.645	L-Pyroglutamic acid	C_5_H_7_NO_3_	130.04996[M + H]^+^	1.15	83.04958[M + H-HCOOH]^+^
30	8.544	Cuminaldehyde	C_10_H_12_O	149.09612[M + H]^+^	0.16	134.07246[M + H-CH_3_]^+^, 105.07019[M + H-CH_3_-CO]^+^, 79.05472[M + H-3CH_3_-CO]^+^
31	14.844	Ursolic acid	C_30_H_48_O_3_	457.36752[M + H]^+^	−0.22	411.35936[M + H-HCOOH]^+^
32	8.791	Taxifolin	C_15_H_12_O_7_	303.05148[M-H]^−^	1.50	285.04037[M-H-H_2_O]^+^, 125.02326[M-H-C_9_H_8_O_4_]^+^
33	8.903	Kaempferol	C_15_H_10_O_6_	287.05478[M + H]^+^	−0.82	213.05362[M + H-2CO-H_2_O]^+^, 153.0179[M + H-C_8_H_6_O_2_]^+^
34	14.344	Quillaic acid	C_30_H_46_O_5_	487.34213[M + H]^+^	0.24	441.33603[M-H-2H_2_O]^+^

## 4 Conclusion

This study aimed to optimize the fermentation process of RRTP using single-factor experiments and Box–Behnken design. The optimal fermentation conditions were determined as a 3:1 ratio of *B. subtilis* to *S. cerevisiae*, a 2:1 mL/g liquid-to-material ratio, a 24 h fermentation time, and a fermentation temperature of 31°C. During fermentation, the TPC, TFC, and TTC of RRTP fermented with single and mixed bacteria were higher than those of the uninoculated group, with the highest TPC obtained from mixed bacteria fermentation. The TPC, TFC, and TTC of the mixed bacteria optimization group were significantly higher than those before fermentation. Ellagic acid and vitamin C contents also increased in different strain fermentation groups and mixed bacteria optimization groups. The mixed bacterial fermentation of RRTP showed enhanced ABTS radical scavenging, DPPH radical scavenging, and total antioxidant capacity (T-AOC) compared to uninoculated bacteria. Under optimal conditions, 34 compounds were identified using UHPLC-ESI-Q-Exactive Plus Orbitrap-MS, compared to only 20 compounds before fermentation. These results suggest that *B. subtilis* and *S. cerevisiae* fermentation can enhance the chemical components and antioxidant activity of RRTP. In conclusion, fermentation of RRTP not only reduces the waste of fruit pomace resources but also provides a way to produce high-value natural additives for feed and agricultural by-products.

## Data Availability

The original contributions presented in the study are included in the article/Supplementary material, further inquiries can be directed to the corresponding author/s.
