# Supplementary material for: Optimization of Rosa roxburghii Tratt pomace fermentation process and the effects of mono- and mixed culture fermentation on its chemical composition
Source: Front Nutr. 2024 Dec 23;11:1494678. doi: 10.3389/fnut.2024.1494678 (PMC11700825; doi:10.3389/fnut.2024.1494678)
Supplement: Supplementary file 1 [file Data_Sheet_1.docx]

Supplementary Material

**Fig.S1**


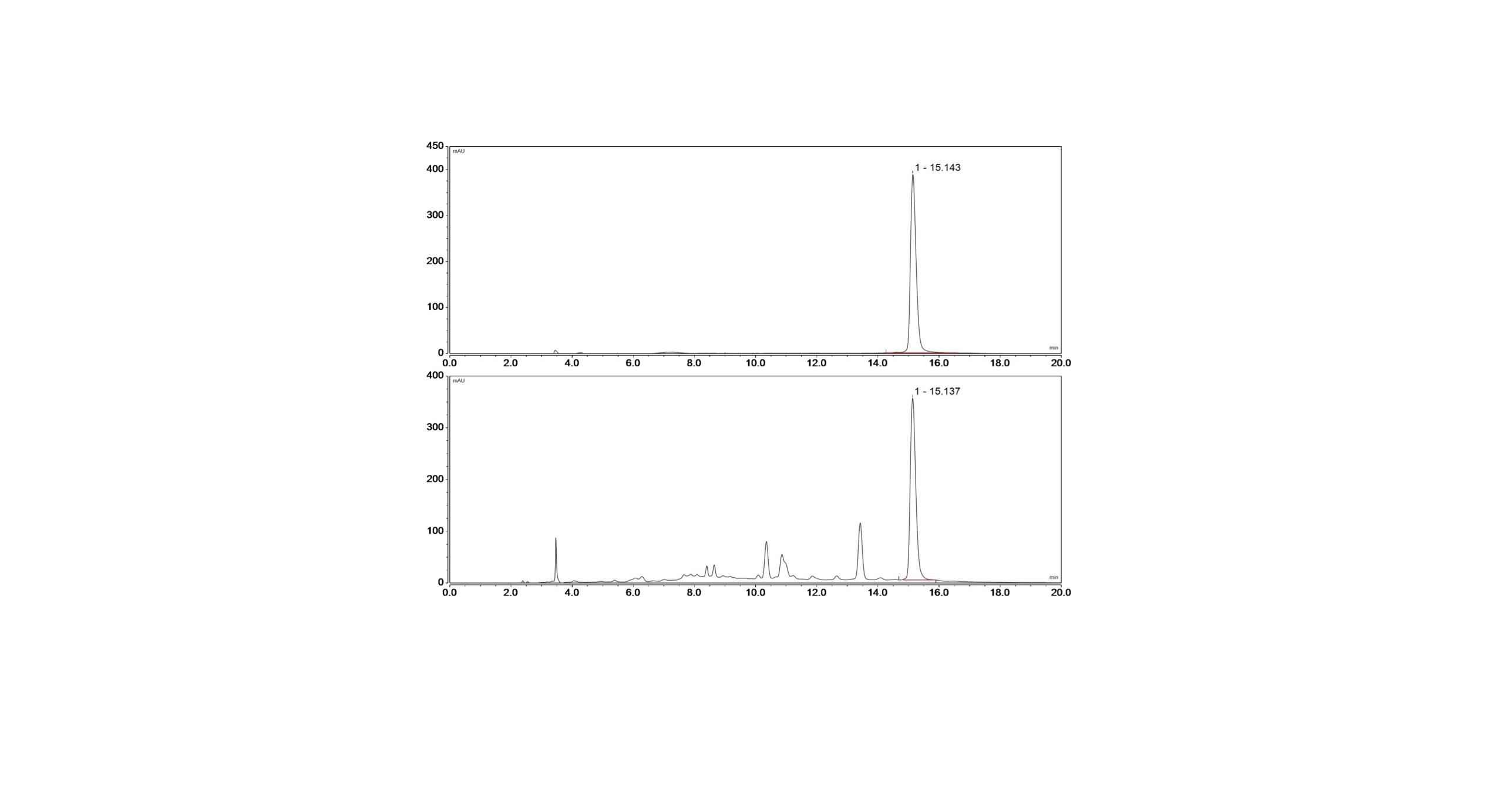


**Supplementary Figure S1.** HPLC chromatograms of ellagic acid reference substance and sample.

**Fig.S2**


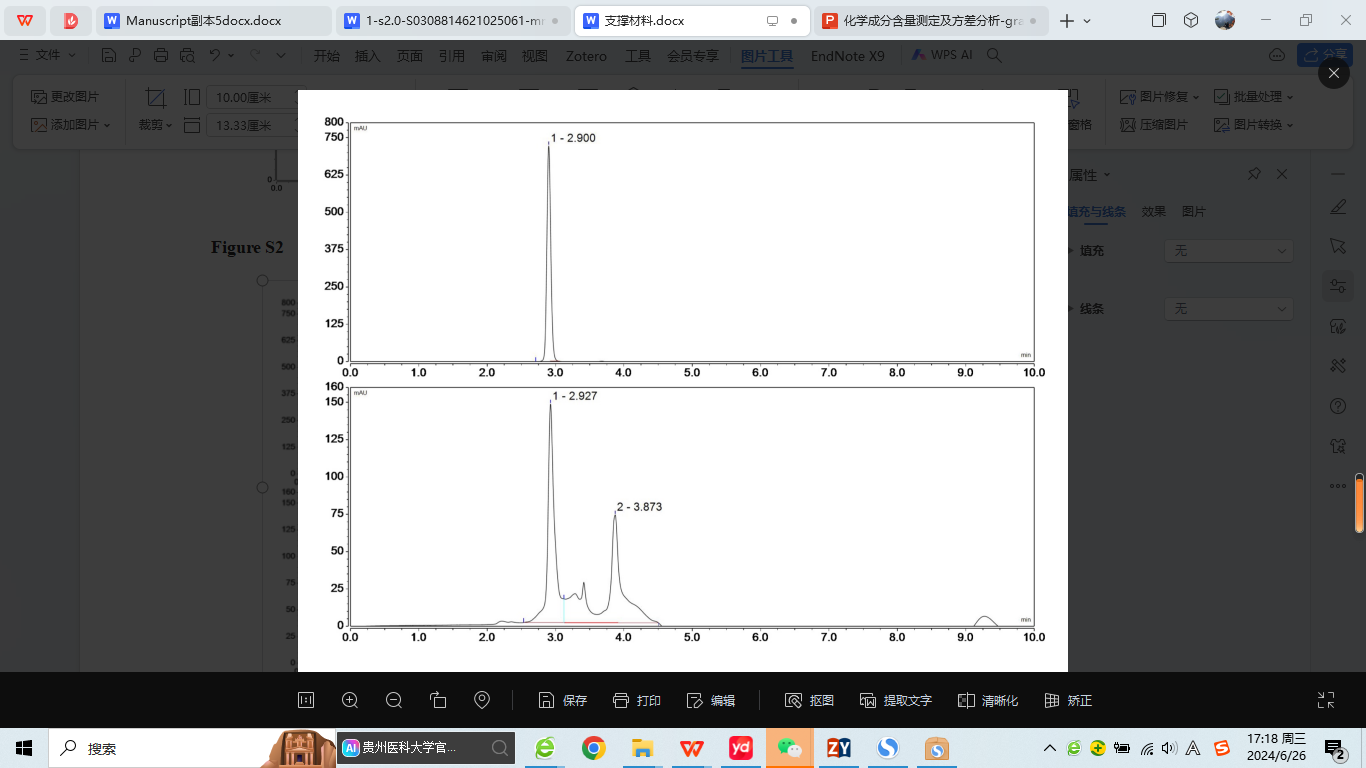


**Supplementary Figure S2.** HPLC chromatograms of Vitamin C reference substance and sample .

**Fig.S3**

**
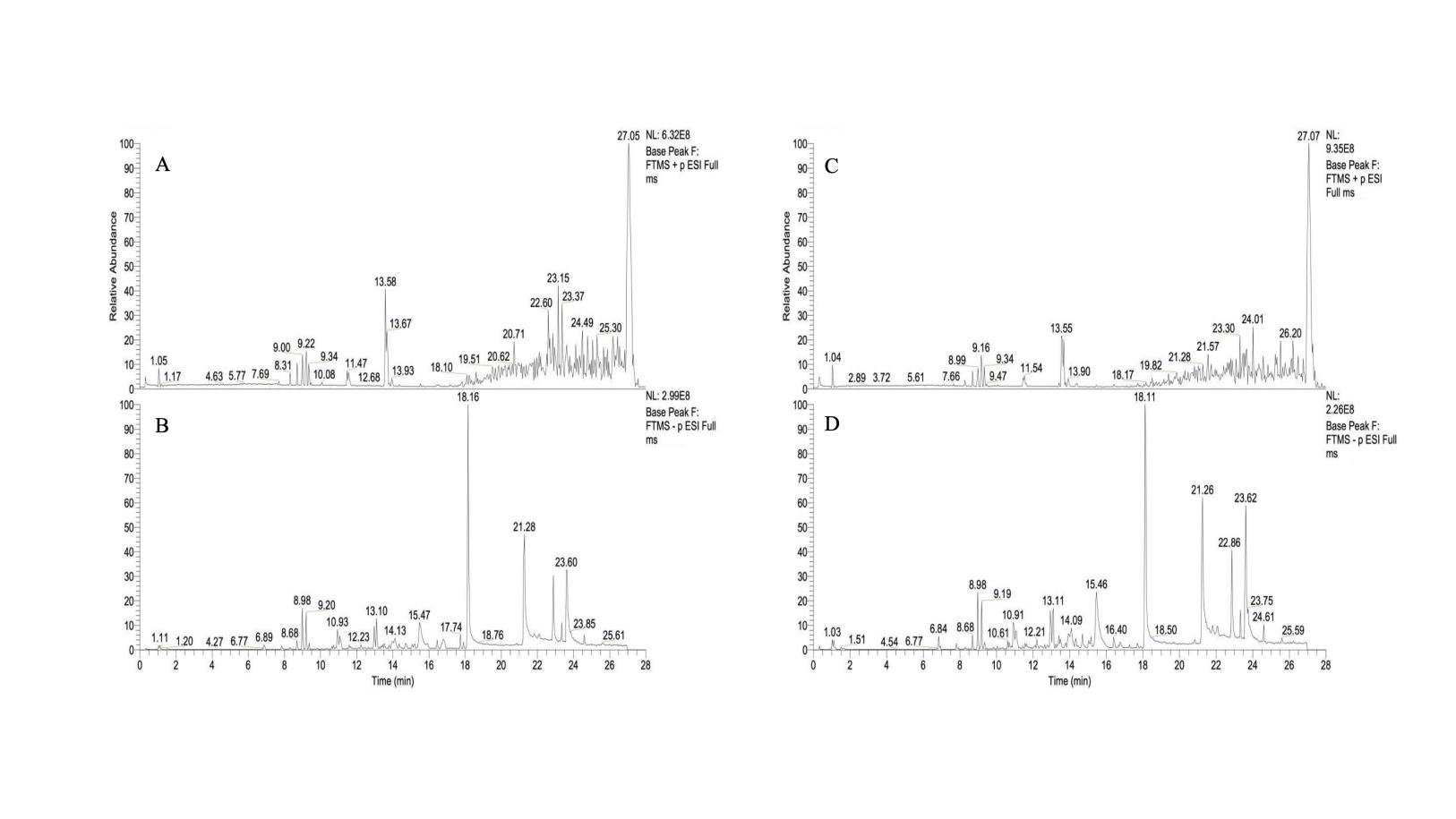
**

**Supplementary Figure S3.** Base peak chromatogram of RRTP in positive (A) and negative ion mode (B) before fermentation. Base peak chromatogram in positive (C) and negative ion mode (D) after RRTP optimization.
